# Structural Model of the Rev Regulatory Protein from Equine Infectious Anemia Virus

**DOI:** 10.1371/journal.pone.0004178

**Published:** 2009-01-12

**Authors:** Yungok Ihm, Wendy O. Sparks, Jae-Hyung Lee, Haibo Cao, Susan Carpenter, Cai-Zhuang Wang, Kai-Ming Ho, Drena Dobbs

**Affiliations:** 1 Department of Physics and Astronomy, Iowa State University, Ames, Iowa, United States of America; 2 Department of Veterinary Microbiology and Preventive Medicine, Iowa State University, Ames, Iowa, United States of America; 3 Bioinformatics and Computational Biology Program, Iowa State University, Ames, Iowa, United States of America; 4 Ames Laboratory–U. S. DOE., Iowa State University, Ames, Iowa, United States of America; 5 Department of Genetics, Development and Cell Biology, Iowa State University, Ames, Iowa, United States of America; 6 Department of Veterinary Microbiology and Pathology, Washington State University, Pullman, Washington, United States of America; University College London, United Kingdom

## Abstract

Rev is an essential regulatory protein in the equine infectious anemia virus (EIAV) and other lentiviruses, including HIV-1. It binds incompletely spliced viral mRNAs and shuttles them from the nucleus to the cytoplasm, a critical prerequisite for the production of viral structural proteins and genomic RNA. Despite its important role in production of infectious virus, the development of antiviral therapies directed against Rev has been hampered by the lack of an experimentally-determined structure of the full length protein. We have used a combined computational and biochemical approach to generate and evaluate a structural model of the Rev protein. The modeled EIAV Rev (ERev) structure includes a total of 6 helices, four of which form an anti-parallel four-helix bundle. The first helix contains the leucine-rich nuclear export signal (NES). An arginine-rich RNA binding motif, RRDRW, is located in a solvent-exposed loop region. An ERLE motif required for Rev activity is predicted to be buried in the core of modeled structure where it plays an essential role in stabilization of the Rev fold. This structural model is supported by existing genetic and functional data as well as by targeted mutagenesis of residues predicted to be essential for overall structural integrity. Our predicted structure should increase understanding of structure-function relationships in Rev and may provide a basis for the design of new therapies for lentiviral diseases.

## Introduction

Equine infectious anemia virus (EIAV) is a member of the lentivirus subfamily of retroviruses, which includes several important pathogens of humans and domestic animals, including HIV-1, the causative agent of AIDS [1]–[3]. Lentiviruses exploit differential and alternative splicing, and overlapping reading frames to generate the proteins necessary for maintaining their life cycles [2], [4]. Fully spliced viral mRNAs produced during the early phase of replication encode regulatory proteins such as Rev and Tat [2]. Incompletely spliced mRNAs give rise to structural proteins, including Pol and Gag that are required for replication and packaging of the viral genome. Rev is a small RNA-binding protein essential for exporting these incompletely spliced mRNAs to the cytoplasm. Export is initiated by the binding of Rev to a specific Rev-responsive element (RRE) in the viral pre-mRNA [2], [5], [6]. The Rev-mRNA complex is then exported to the cytoplasm by interaction of the nuclear export signal (NES) of Rev with CRM1 (or exportin 1), a component of the cellular nuclear export machinery [7]–[10]. Rev itself shuttles back into the nucleus using the interaction of its nuclear localization signal (NLS) with cellular nuclear import proteins [11]–[13]. Mutations in either the NES or NLS can abolish Rev function and block the production of infectious virus [14]–[16]. Although Rev has long been viewed as a promising target for antiviral therapies, the development of drugs that inhibit Rev function has been hindered by a lack of information regarding Rev structure. The principal stumbling block to structure determination is the tendency of Rev to aggregate at concentrations needed for crystallization or solution NMR studies [17]–[19]. In HIV-1, beyond a critical threshold of about 6 uM, Rev polymerizes into regular, unbranched filaments [18]. Recent solid state nuclear magnetic resonance (NMR) study on HIV-1 Rev filaments and Rev-RNA co-assembly suggested that although their morphologies are qualitatively different, protein conformations in each assembly are the same, supporting the previous helix-loop-helix structural model [20]. However, so far, the only available high-resolution structure is an NMR solution structure of a 23 amino acid fragment of Rev bound to a 34-nucleotide RRE-RNA fragment [21].

**Figure 1:**
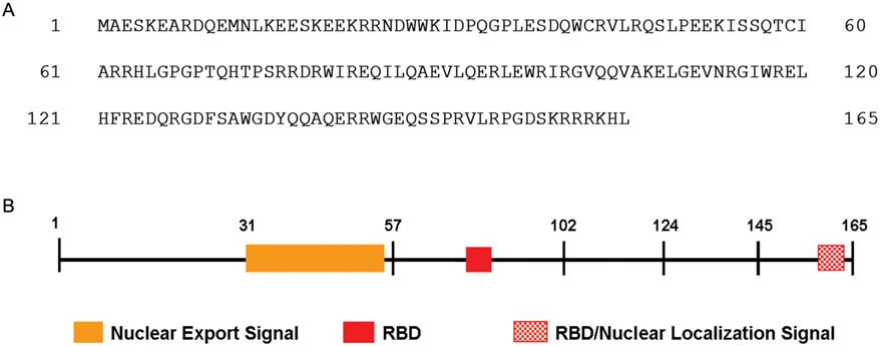
The sequence and functional domain organization of ERev. (A) The amino acid sequence of ERev variant R1: a representative sequence used in the threading study. (B) Functional domain organization of ERev.

Previously, we have investigated the role of genetic variation in EIAV persistence and pathogenesis [22]–[25]. One of the most variable regions in the EIAV genome is in the region where the Rev gene overlaps sequences encoding the cytoplasmic portion of the transmembrane protein [23]. Recent studies have focused on mapping the functional domains of EIAV Rev (ERev) shown in Fig. 1 [25], [26] and determining the effect of genetic variation on Rev activity [24], [27]. As part of those efforts, we sought to develop a structural model of ERev.

We used a combined computational and experimental approach to propose and evaluate a three-dimensional structural model of the ERev protein. We adopted a structural threading scheme that focuses on structural similarities with minimal reliance on sequence homology, a strategy that is useful when sequence similarity is weak or undetectable (sequence identity <25%) [28]. We evaluated the validity of the proposed ERev model in the context of previously published genetic and functional data and tested specific predictions of the model by assaying the effects of amino acid substitutions on Rev activity in transient expression assays. These approaches, together with a comparative analysis of the EIAV and FIV Rev structures, support the validity of the proposed three-dimensional structure of ERev. The model provides insight into the structural basis of Rev function that may increase understanding of CRM1 dependent export proteins in a number of virus species.

## Results and Discussion

### Structural model of EIAV Rev

In the threading studies, fragments of the ERev protein have been threaded against 13,391 representative structures in the structural template library, and the model structures were obtained from the top scoring members of the top five families using MODELLER [29], [30] (see Materials and Methods).

**Figure 2:**
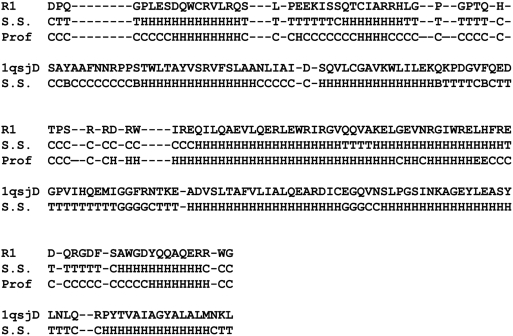
The sequence and secondary structure alignments based on threading. The sequence alignment between the ERev (R1) and the best scoring template (1qsjD), along with the secondary structures obtained from the model (S.S. of R1), template structure (S.S. of 1qsjD), and that predicted using Prof.

The results of the threading on top five template structures and their secondary structure composition in each model are shown in Table 1. The best score of 37 was obtained from the threading of a fragment of ERev corresponding to amino acids 31–145 against the region corresponding to amino acids 1,075–1,200 of the structure of an N-terminally truncated rat serum complement C3d fragment [31] (PDB code 1qsj chain D). The family to which 1qsjD belongs had 6 members and all of them gave the threading score greater than the threshold. The next four templates gave much lower threading scores, ranging from 28 to 31. The secondary structure composition of the top model was the average of the predicted secondary structure composition by Prof and Psipred. In two of the four alternative folds (template 1iar and 1hc1), the secondary structure composition from the model was also the average of the predicted secondary structure using Prof and Psipred. In the other two alternative models, the secondary structure composition from the model was significantly different from those of predicted versions. In Fig. 2, the sequence alignment between the ERev and the best scoring template is shown along with the secondary structures obtained from the model, template structure, and that predicted using Prof. The secondary structure obtained from the model agrees well with the predicted one. With the significant threading score as well as a good agreement with the predicted secondary structure, the top model was selected for further analysis.

**Table 1:** Results of the threading on top five template structures and their secondary structures.

Aligned region (a.a.)	Template PDBID	Threading score	S.S. using model (α, β, coil)^a^	S.S using Prof (α, β, coil)	S.S using Psipred (α, β, coil)
31–145	1qsj	37	(59, 0, 41)	(54, 2, 44)	(63, 0, 37)
31–150	1iar	31	(57, 0, 43)	(52, 1, 47)	(61, 0, 39)
1–140	1occ	28	(46, 0, 54)	(56, 2, 42)	(60, 2, 38)
36–155	1hc1	28	(58, 0, 42)	(52, 1, 47)	(61, 2, 37)
66–145	1bxr	28	(53, 0, 47)	(60, 2, 38)	(70, 0, 30)

a Percentage of alpha helix, beta strand, and coil.

**Figure 3:**
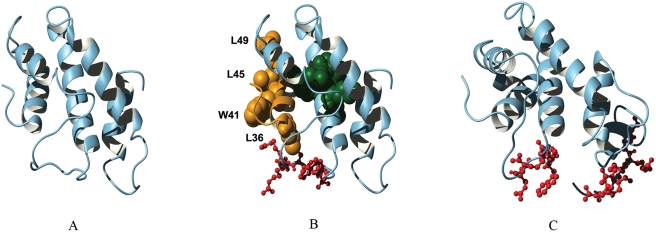
Structural model of ERev. (A) The model of the truncated Rev exon2 (a.a. 31–145). (B) Known functional domains are mapped on the model structure of the Rev exon2. The NES domain is colored in orange with four hydrophobic residues critical for export activity shown in space-fill. The RRDRW RNA-binding motif is depicted in red ball-and-stick. The ERLE motif appears in green space-fill. (C) The model of the full-length Rev exon2 (a.a. 31–165). Basic residues in RRDRW and KRRRK motifs are depicted in red ball-and-stick.

A full atomistic model of the top model is shown in Fig. 3A. The region containing amino acids 31–145 consists of five helices, four of which are configured in a four-helix bundle (helix 1 to helix 4). The first 30 residues of ERev, corresponding to exon 1, were not modeled in our experiments; however, exon 1 sequences are not required for Rev function [11]. A structure of the full-length Rev exon 2 (a.a. 31–165), including the C'terminal RNA-binding/NLS region, yielded a lower threading score against the same template (PDB code 1qsj). The major difference in the alignment occurred starting from amino acid position 95 and there was shift in the alignment in the fifth helix. The model structure for the full-length exon 2 Rev sequence is shown in Fig. 3C. Despite the difference in the alignment in the fifth helix, the overall topology of the full-length Rev exon 2 is very similar to that of the truncated version (a.a. 31–145) shown in Fig. 3A.

### EIAV Rev structural model is consistent with genetic analyses of functional domains

To determine if the structural model was consistent with previously published genetic data of Rev functional domains, we mapped the known functional and/or essential domains of ERev onto the three-dimensional structural models of truncated and full-length Rev exon 2 (Fig. 3B and C). The leucine-rich nuclear export signal (NES) is located in the first helix of our model (Fig. 3B). The NES interacts with CRM1 to effect export of incompletely spliced RNA to the cytoplasm [8], [10], and the sidechains of the four hydrophobic residues required for NES activity (L36, W41, L45, L49) are directed outward in our model. The solvent accessible surface area (ASA) associated with three of these four hydrophobic residues is very high (26.6–57.3%), which is consistent with their role in mediating protein-protein interaction with CRM1. L36 is highly buried (ASA = 0.9%) due to its contact with L90, but it is feasible that this contact may be disrupted upon Rev-RNA binding.

The RNA-binding domain of ERev is comprised of two short, discontinuous motifs: RRDRW (a.a. 76–80) in the central region of ERev, and the C'terminal KRRRK (a.a. 159–163) [26]. The KRRRK motif is also required for nuclear import [26]. The arginine-rich RRDRW motif, represented in red ball-and-stick in Fig. 3B and C, is located in a solvent-exposed loop connecting the second and third helices. The KRRRK motif is juxtaposed with arginine-rich RRDRW motif on the surface of the folded structure corresponding to the complete exon 2 Rev sequence (Fig. 3C). Although the full length exon 2 model structure containing the KRRRK region is not well justified by significant threading score, it suggests the possibility that RRDRW and KRRRK motif together may form a single continuous arginine-rich motif on the surface of Rev structure, making the interaction with RNA favorable.

Mutations and/or deletions in the central regions of ERev exon 2 have been shown to significantly reduce Rev nuclear export activity [8], [25], [27], [32]. Alanine substitution of an ERLE motif in the central region (a.a. 93–96) abrogated Rev nuclear export activity, and this motif was previously proposed as an RNA binding domain [8], [32]. More recently, however, we found no decrease in RNA binding when alanine was substituted for only the charged residues in the ERLE motif [26]. In contrast to RRDRW, the ASA calculations based on our model indicate the ERLE motif is very much buried in the protein core of ERev (Table 2). In addition, two residues in this motif are predicted to make contacts important for stabilization of the structure: L95 makes several inter-helical hydrophobic contacts (see below) and the R94 forms a salt bridge with D39. When considered with our experimental RNA binding studies [26], the structural model supports a role for ERLE in maintaining the structural integrity of ERev essential for Rev function *in vivo*. In addition, these results indicate that appropriately folded structure of Rev is required for RNA binding.

**Table 2:** Solvent accessible surface area (ASA) associated with residues in the RRDRW and the ERLE motifs.

Residue (RRDRW)	ASA (%)	Residue (ERLE)	ASA (%)
R76	32.8	E93	10.5
R77	65.6	R94	0.0
D78	19.5	L95	0.3
R79	62.1	E96	12.2
W80	26.4		

ASA values were calculated using MOLMOL.

### The structural model is supported by targeted mutagenesis of critical residues

**Figure 4:**
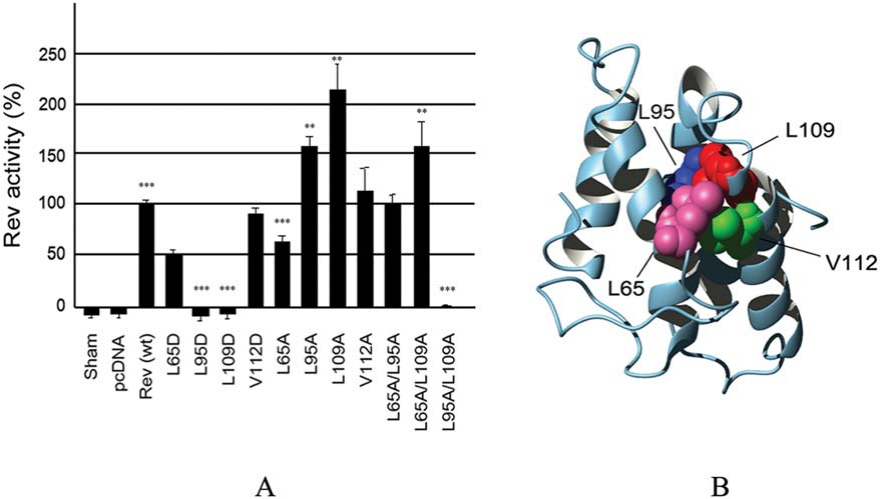
Nuclear export activities of ERev mutant proteins. (A) The level of Rev nuclear export activity associated with single Ala, Asp mutations, and double Ala mutations. The results are expressed relative to wildtype Rev variant R1, and represent the mean activity of at least six independent transfections, ±standard error. Variants that differed significantly from the activity of R1, according to a two-tailed t-test, are indicated by asterisks, *p<0.05; **p<0.005; ***p<0.0005. (B) Mutation sites are displayed in space-fill in the truncated Rev exon2 model structure.

In order to further validate the model structure, we sought to identify specific residues expected to be most important for the structural integrity of ERev. For this, we obtained inter-helical hydrophobic contact information for each residue in ERev model structure. Inter-helical contact analysis revealed that three residues, L65, L95, and L109, participate in more than three inter-helical hydrophobic contacts in the model structure. Interestingly, L95 is located within the ERLE motif discussed above. The inter-helical contact and ASA data associated with these residues are shown in Table 2, and the three residues are represented in space-fill in Fig 4B. The ASA values for L95 and L109 are close to 0%, and that of L65 is 5.7%. This suggested that L95 and L109 might make the most important hydrophobic contact to stabilize the Rev structure and the structural importance of L65 would be of lesser significance.[Table pone-0004178-t003]

**Table 3 pone-0004178-t003:** Inter-helical hydrophobic contacts and solvent accessible surface area in the truncated model structure of ERev.

Residue	Number of inter-helical hydrophobic contacts	ASA (%)
L65	3	5.7
L95	3	0.3
L109	4	0.2

To test this hypothesis, mutations were introduced at each of the three sites, and the effect of mutations was assessed using CAT-based transient expression assay. The mutation strategy was based on that introduced by Thomas and coworkers to test the structural roles of selected amino acids in HIV-1 Rev [33]. In order to test the structural importance of selected residues, they first mutated those individual residues to aspartic acid, which are incompatible with formation of buried hydrophobic contacts. The mutation effect on Rev structure, which is destabilization of the protein structure, is manifest as a dramatic reduction in Rev activity in CAT-based assays. As a second step, in order to directly test residue contacts, they introduced both individual single alanine mutations and simultaneous double alanine mutation to residue pairs predicted to be in contact. Because proteins have inherent flexibility, the cavity created by single alanine mutation will be filled by the compensatory rearrangement of surrounding residues. In contrast, the cavity created by simultaneous double alanine mutation cannot be compensated, which severely destabilizes the protein structure and results in reduction in Rev activity. The analysis of such double mutations should indicate which predicted inter-helical contacts are most important for stabilizing Rev structure and maintaining full Rev activity. Using the same mutational strategy, mutations were introduced into the ERev protein and the effect of mutations was evaluated in a Rev nuclear export activity assay (Fig 4A). As expected, Asp mutations on L95 and L109 significantly reduced nuclear export activity, indicating that these residues play critical roles in stabilizing the ERev structure. A less critical role for L65 was supported by maintenance of 50% of Rev activity in L65D. Further, disruption of the hydrophobic contact between L95 (within the ERLE motif in helix 3) and L109 (within helix 4) introduced by L95A/L109A double Ala mutation completely abrogated nuclear export activity. This suggests that inter-helical contact between these two amino acids is critical for maintaining a functional Rev structure. In contrast, the L65-L95 and L65-L109 contacts appear to have negligible impact on Rev activity, indicating that these two contacts are of less significance for the Rev structure. L65A/L109A mutation showed no decrease, but rather an increase in Rev activity by 49% compared with R1. It is likely that the increase mainly occurred due to the stabilizing effect of L109A mutation (see discussion below). The decrease of the Rev activity upon L65A/L95A mutation (5%) was statistically insignificant.

Surprisingly, we observed significant increases in Rev activity upon single Ala mutations at L95 and L109. The contact data analysis revealed that L109 makes an intra-helical hydrophobic contact with V112 in addition to four inter-helical hydrophobic contacts with L65, L95, I99, and V102 (see Table S1 in Supporting Information). Notably, three out of five contact residues for L109 are beta-branched hydrophobic residues (I99, V102, and V112). L95 makes an intra-helical hydrophobic contact with L91 and three inter-helical hydrophobic contacts with L65, L109, including a contact with beta-branched V112. Beta-branched residues have limited sidechain rotational degree of freedom due to the steric hindrance with i-3 and i-4 carbonyl oxygens in g^+^ rotamer state, and thus can cause distortion in local helix backbone. The effects of beta-branched residues on alpha-helix stability have been intensively studied and shown to destabilize helix propensity [34], [35]. Contacts involving many beta-branched hydrophobic residues in the crowded protein core may generate strain in helices, destabilizing the structure. Mutations that relieve this strain might be expected to increase Rev activity. In order to test the strain effect on protein stability by beta-branched residues, we introduced Ala mutation into V112. The V112A mutation increased Rev activity to 117% of R1 Rev activity (Fig. 4A), indicating that releasing strain can actually increase Rev activity. A similar mechanism may account for the increased Rev activity observed in L95A and 109A mutants.

The targeted mutagenesis of ERev, however, did not support four alternative model structures (Table S2). L65, L95, and L109 in alternative models either reside in a loop or make insignificant number of inter-helical hydrophobic contacts, ranging from 0 to 2, if they reside in helices. While the total number of inter-helical hydrophobic contacts made by these three residues was 10 in the top model, those in the alternative models were in between 1 to 4. This, together with the significantly higher threading score of the top model and good agreement with the predicted secondary structure supports our predicted model.

Our structural model provides new information on the structural features of the NES domain that interacts with CRM1 as well as the RNA binding domain in the context of the entire structure of the Rev exon 2. CRM1-dependent nuclear export is a key step in the replication of many viruses, including retroviruses and influenza viruses [36], [37]. Our structural model will enhance our studies on the structural basis of protein-protein interactions required for successful virus replication and will provide aid in the design of effective antiviral drugs. In addition, our model provides structural information on the “hyper-variable” or “non-essential” region that was first identified by Belshan and co-workers [23]. The hyper-variable region, located in the loop connecting the fourth and the fifth helices in our model, has been shown to be dispensable for Rev activity, however, the high numbers of mutations in this area can result in significant fluctuations in levels of Rev activity *in vitro*. Our model provides structural basis on the ability of the non-essential region in ERev to withstand a variety of genetic variation. Identifying such non-essential loops in genes of overlapping reading frames based on the model is very useful in our ability to gain better understanding and control of the virus.

### EIAV and FIV Rev share similar structural features

**Figure 5:**
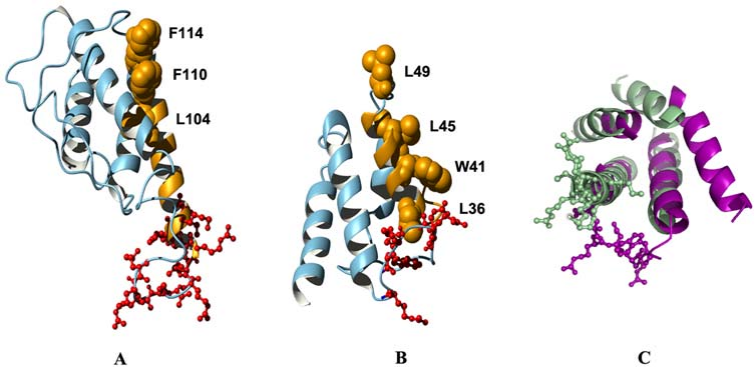
Predicted structural model of FIV Rev protein compared with that of ERev. (A) FIV Rev model structure corresponding to the region a.a. 16–145. The NES domain is shown in orange, with three hydrophobic residues critical for export activity shown in space-fill. Arginine and lysine residues in a highly basic region that may play a role in RNA binding and nuclear import are shown in red ball-and-stick. (B) ERev model structure containing helix 1 to helix 4. Four hydrophobic residues in NES domain are shown in orange space-fill representation, and arginine residues in RRDRW motif are shown in red ball-and-stick. (C) The structure alignment between the FIV (green) and the ERev (purple) models. For clarity, only the first four helices of the ERev model are shown. Also, all loops except the region between the RRDRW motif and the third helix of the ERev model are not shown.

In addition to ERev, we also used our threading algorithm to model the structure of Rev proteins from other lentiviruses, including FIV Rev. For the FIV Rev, we obtained a significant threading score for the region corresponding to amino acids 16–145 against the structure of interleukin 4 structural mutant [38]. Interestingly, the modeled region of FIV Rev forms a four-helix bundle with structural similarity to the region of helix 1 to helix 4 of ERev model structure (Fig. 5). Using Dali structure comparison method [39], the structural similarity between the FIV and the first four helices of EIAV Rev model structures was significant (CA RMSD = 2.8 Å). Although FIV Rev is not as well characterized as ERev, the location of NES domain in our FIV Rev model is similar to that of ERev. The FIV Rev NES domain (a.a. 97–120) is unusually large [9], [10] and in our model extends from helix 3 through the loop connecting helix 3 and 4. Amino acids known to be critical for protein-protein interaction with CRM1[10] are facing outward. A highly basic domain (a.a. 83–94) that could play a role as a RNA binding domain, is located in the region including the C-terminal part of the loop connecting the second and third helices and the N-terminal part of the third helix. Arginine and lysine residues in this region are facing outward with high solvent accessibility (ASA between 22–67%), similar to the situation in ERev. The similarities in overall structure and spatial organization of functional domains in ERev and FIV Rev occurred despite the fact that they share insignificant sequence similarity (BLAST alignment E-value = 6.4). More extensive comparative structural threading analysis, applied to both primate and non-primate lentiviruses, may provide additional insight into common structural features important for Rev function.

## Materials and Methods

### Structural threading

In threading, a target protein sequence is aligned with a library of structural templates from known structures in the Protein Data Bank (PDB), and a sequence-to-structure alignment for each template is evaluated to identify the fold with best “fit” [28], [40], [41]. In our approach [28], candidate structures are represented by contact matrices, following the work of Miyazawa and Jernigan [42]. The position of an amino acid sidechain is defined by the average heavy atom position, referred to as the center of position (COP). Two residues, i and j, are defined to be in contact (C_ij_ = 1), if the distance between their COP's is less than or equal to 6.5Å, and not in contact (C_ij_ = 0), otherwise. For threading alignment, initial profiles representing the template structures are generated from the first four eigenvectors of the contact matrices. To predict the structure for a target protein, its sequence is threaded against all the structures in the structural template database in search of a structure with a significant “fit” to the sequence. The structural template database for threading consisted of 13,391 representative structural domains selected from the Astral 1.61 domain library, covering 1,939 families of SCOP domains [43], [44]. Redundancy of the database was reduced by including only 20 representative structures for any family with more than 20 members. Otherwise, all structures in each family were included in the database. The strength of each alignment is determined by a scoring function consisting of a sum of all residue-residue contacts. Hydrophobic strengths are evaluated using the Li, Tang and Wingreen parameterization [45] of the Miyazawa-Jernigan matrix [42]. Local secondary structure preference is incorporated by enhancing the threading score if the predicted secondary structure of the target protein sequence matches that of the template structure. Secondary structure assignments for the template structures were generated from their PDB coordinates using Stride software [46].

The input to the threading process is the sequence and predicted secondary structure of the ERev protein. Although there is a high rate of Rev variation in vivo, ERev amino acid variants share over 92% sequence similarity among themselves [27]. Rev variant R1, which was originally identified as the dominant variant in a horse experimentally infected with the virulent EIAV_Wyo2078_ [23], was selected as a representative ERev protein sequence. Secondary structure of the full length R1 Rev sequence was predicted using three different methods, PSIPRED [47], PROF [48], and Sam-T99. The final secondary structure profile of the target sequence was assigned such that, for each residue in the sequence, if the secondary structure prediction by different servers agreed, the consensus of the prediction was assigned as the secondary structure for the residue, otherwise, the secondary structure was left unassigned. Prior to threading, the ERev sequence was fragmented into overlapping segments with a minimum length of 60 residues and with starting positions every 5 residues. The length of the fragments was also varied, from 60 a.a. to full length (165 a.a.), in 5 residue increments. All fragments generated in this manner were threaded against the 13,391 representative structures in the structural template library and the threading score for each fragment was calculated. The threading score, originally termed as the relative score in Cao et al. [28], is defined by the difference between the raw threading score of the native sequence and the average of those of randomly shuffled sequences. The significance of the threading was determined on the family basis: if over 75% of the members in a family give the threading scores above threshold, the alignment between the sequence and the template structures from the family was considered significant. Model structures from the top five families were obtained from the alignments with the top scoring member of the family. Details of the threading scheme are provided in Cao et al. [28]. After threading, full atomistic models of the Rev protein were generated from the template and alignment obtained from the threading studies using the MODELLER [29], [30], and NEST software tool incorporated in JACKAL 1.5 [49].

### Characterization of Rev structural mutants

Mutations predicted to specifically disrupt inter-helical contacts or tertiary structure of ERev were introduced into the Rev expression vector pcH21_SL_ using PCR-based site-directed mutagenesis^25^. All mutations were confirmed by sequencing and Rev nuclear export activity was quantified in transient transfection assays using chloramphenicol acetyltransferase (CAT) reporter plasmids containing EIAV RRE, as previously described [24]. Plasmids encoding wild-type Rev or Rev mutants were co-transfected into HEK 293T/17 cells (293T, ATCC CRL-11268) with 0.2 µg of EIAV RRE reporter plasmid and 0.2 µg of pCH110. Each experiment included a sham group that contained no reporter plasmid, but an additional 0.2 µg of pUC19. Two days post-transfection, cells were harvested, resuspended in 0.3 ml 0.25 M Tris (pH 7.5), lysed by freeze/thawing, and assayed for β-galactosidase activity to normalize CAT assays for transfection efficiency. Normalized lysates were assayed for CAT levels using a CAT ELISA kit (Roche Applied Science). Each mutant was assayed in triplicate and the results represent at least six independent transfections normalized to wild-type Rev. Results were analyzed using two-tailed Student's t-test assuming unequal variance among groups to detect significant differences between mutants and wild-type Rev.

## Supporting Information

Table S1 Inter-helical and intra-helical contacts associated with selected residues in the truncated Rev exon2 model.
(0.03 MB DOC)

Table S2 Number of inter-helical hydrophobic contacts associated with selected residues in the top five model structures.
(0.03 MB DOC)
