# Supplementary material for: Structural Model of the Rev Regulatory Protein from Equine Infectious Anemia Virus
Source: PLoS One. 2009 Jan 12;4(1):e4178. doi: 10.1371/journal.pone.0004178 (PMC2613556; doi:10.1371/journal.pone.0004178)
Supplement: Table S1 — Inter-helical and intra-helical contacts associated with selected residues in the truncated Rev exon2 model. (0.03 MB DOC) [file pone.0004178.s001.doc]

Table S1. Inter-helical and intra-helical contacts associated with selected residues in the truncated Rev exon2 model.

| Residue | Inter-helical hydrophobic contacts | Intra-helical hydrophobic contacts | All contacts |
| --- | --- | --- | --- |
| L65 | L95, L109, V112 |  | A61, L95, A106, E108, L109, V112 |
| L95 | L65, L109, V112 | L91 | A61, L65, L91, L109, V112, N113 |
| L109 | L65, L95, I99, V112 | V102 | A61, L65, L95, I99, V102, Q103, A106, V112, N113 |
