# Supplementary material for: Structural Model of the Rev Regulatory Protein from Equine Infectious Anemia Virus
Source: PLoS One. 2009 Jan 12;4(1):e4178. doi: 10.1371/journal.pone.0004178 (PMC2613556; doi:10.1371/journal.pone.0004178)
Supplement: Table S2 — Number of inter-helical hydrophobic contacts associated with selected residues in the top five model structures. (0.03 MB DOC) [file pone.0004178.s002.doc]

Table S2. Number of inter-helical hydrophobic contacts associated with selected residues in the top five model structures.

| Aligned region  (a.a.) | Template  PDBID | L65 | L95 | L109 |
| --- | --- | --- | --- | --- |
| 31-145 | 1qsj | H (3)^a^ | H (3) | H (4) |
| 31-150 | 1iar | L (0) ^b^ | H (0) | H (1) |
| 1-140 | 1occ | L (2) | L (0) | L (1) |
| 36-155 | 1hc1 | L (0) | H (1) | L (0) |
| 66-145 | 1bxr | N/A | H (2) | L (2) |

^a^ L65 resides in a helix in the model structure making three hydrophobic contacts with other residues in other helices.

^b^ L65 resides in a loop in the model structure making no hydrophobic contacts with other residues in other helices.
